# Inhibition of O-GlcNAc transferase sensitizes prostate cancer cells to docetaxel

**DOI:** 10.3389/fonc.2022.993243

**Published:** 2022-11-10

**Authors:** Mingyue Xia, Shuyan Wang, Yannan Qi, Kaili Long, Enjie Li, Lingfeng He, Feiyan Pan, Zhigang Guo, Zhigang Hu

**Affiliations:** Jiangsu Key Laboratory for Molecular and Medical Biotechnology, College of Life Sciences, Nanjing Normal University, Nanjing, China

**Keywords:** OGT, chemotherapy, docetaxel, miR-140, prostate cancer

## Abstract

The expression of O-GlcNAc transferase (OGT) and its catalytic product, O-GlcNAcylation (O-GlcNAc), are elevated in many types of cancers, including prostate cancer (PC). Inhibition of OGT serves as a potential strategy for PC treatment alone or combinational therapy. PC is the second common cancer type in male worldwide, for which chemotherapy is still the first-line treatment. However, the function of inhibition of OGT on chemotherapeutic response in PC cells is still unknown. In this study, we show that inhibition of OGT by genetic knockdown using shRNA or by chemical inhibition using OGT inhibitors sensitize PC cells to docetaxel, which is the most common chemotherapeutic agent in PC chemotherapy. Furthermore, we identified that microRNA-140 (miR-140) directly binds to *OGT* mRNA 3′ untranslated region and inhibits OGT expression. Moreover, docetaxel treatment stimulates miR-140 expression, whereas represses OGT expression in PC cells. Overexpression of miR-140 enhanced the drug sensitivity of PC cells to docetaxel, which could be reversed by overexpression of OGT. Overall, this study demonstrates miR-140/OGT axis as therapeutic target in PC treatment and provides a promising adjuvant therapeutic strategy for PC therapy.

## Introduction

According to the global Cancer Statistics 2020, PC is the fourth most common type of cancer worldwide and the second leading cause of cancer-related deaths among men (1). There are more than 1.2 million new prostate cancer diagnoses annually and more than 350,000 deaths worldwide (2). Depending on the stage or classification (androgen dependent or castrate resistant), different treatment options, such as androgen deprivation therapy (ADT), radiotherapy, or chemotherapy may be considered for PC management (3). Following ADT, most hormone-sensitive patients develop castration-resistant prostate cancer (CRPC), which is the leading cause of death (4). Clinical data show that 10–20% of patients with metastasis prostate cancer develop CRPC within 5 years of follow-up, and the median survival since the development of castration resistance is approximately 14 months (range 9–30 months) (5). Additionally, patients with non-metastatic CRPC are at higher risk of disease progression. Approximately 15–33% of patients develop metastasis within 2 years, increasing the burden of mortality in this population (6, 7). Until 2004, mitoxantrone plus low-dose prednisone was commonly used in CRPC. Clinical trials of docetaxel-based chemotherapy showed for the first time an improvement in overall survival (OS) (8). Docetaxel inhibits microtubule depolymerization, thereby inhibiting cell mitosis and inducing apoptosis events. In the TAX327 trial treatment with docetaxel every 3 weeks, prolonged survival from 16.5 months to 18.9 months compared with mitoxantrone (8).The updated analysis from 2008 showed an increase in median OS to 19.2 months in the docetaxel-treated group (9). Despite these advances, the use of chemotherapy in PC is still limited by the development of chemoresistance and significant toxicities (10). There is an urgent need not only for new therapeutic options, but also for novel combination strategies (4, 11).O-GlcNAcylation is a post-translational modification that transfers β-linked N-acetylglucosamine (GlcNAc) to serine or threonine residues by OGT. In contrast, the O-GlcNAcase (OGA) remove O-GlcNAc from the protein, thus the whole process is reversible and dynamic (12, 13). There is growing evidence that hyper-O-GlcNAcylation has become a general feature of cancer progression and metastasis (14–17). Cancer cells elevate total O-GlcNAcylation by increasing OGT and/or decreasing OGA levels. Many cancer types display elevated O-GlcNAcylation and abnormal OGT and OGA expression. For example, several studies have shown that PC cell lines and patient samples contain elevated levels of OGT and O-GlcNAc compared to normal counterparts (18–20). Various research groups have shown that OGT and O-GlcNAc levels increase during the progression of breast cancer which correlates with the histological grade of the tumor (21, 22). Similar trends have been observed in a number of cancer types including colon (23, 24), bladder (25), hepatocellular carcinoma (26), leukemia (27, 28), lung (23, 29);and pancreatic cancer (30). As the overall elevations in O-GlcNAcylation is recognized as a common feature of cancer cells, OGT is positioned as a novel therapeutic target with potent anti-glycolytic activity (17). Reduction of O-GlcNAcylation in cancer cells inhibits oncogenesis (17). Itkonen et al. showed that inhibition of OGT activity inhibited the proliferation of PC cells. And simultaneous inhibition of OGT and GPT2 (alanine aminotransferase) inhibited PC cell viability and growth rate, and additionally activated a cell death response (31). Previous studies showed that the expression level of O-GlcNAc in PC cells is associated with poor prognosis in PC patients and may enhance the proliferation and invasion of tumor cells (16). Ortiz-Meoz et al. reported that OSMI-1,a small molecule inhibitor targeting OGT, inhibited OGT activity in a dose-dependent manner (32).

Recent studies have shown that microRNAs (miRNAs) emerge as novel biomarkers and potential therapeutic targets in the treatment of prostate caner (33). MiRNAs, are small non-coding RNAs (ncRNAs) that can regulate gene expression by binding to target mRNAs (34). MiRNA have been found to regulate important cellular functions, including apoptosis, proliferation, cell cycle, differentiation, stem cell maintenance and metabolism (35). Recent studies have reported that the dysregulation of miRNA expression profiles is associated with PC and emerged as a novel biomarker and potential therapeutic target for the treatment of PC (33, 36, 37). According to the report, miR-877-5p may act as a suppressor in PC and reduces cancer cell proliferation, migration and invasion by targeting FOXM1 (38). miRNA-125b has been attributed as an oncogene in the pathogenesis of PC through down regulation of Bak1 (39). These indicate that miRNA may serve as the effective biomarkers and therapeutic target for treating PC patients.

In our study, we detected whether targeting OGT altered the cellular response to docetaxel chemotherapy. We showed that inhibition of OGT enhanced chemotherapeutic sensitivity of PC cells to docetaxel. We further used bioinformatic analysis to identify miR-140 as a potential miRNA targeting OGT. Indeed, we demonstrated that miR-140 inhibited OGT expression by directly targeting the OGT 3′UTR. In addition, we found that overexpression of miR-140 inhibited OGT expression and increased the sensitivity of PC cells to docetaxel, which could be reversed by overexpression of OGT. Our findings provide a novel strategy for PC therapy.

## Materials and methods

### Cell culture

The human DU145 cells, PC3 cells, and normal human prostate epithelial cells (Wpmy-1) were obtained from the American Type Culture Collection. Subsequently, they were grown in 1640 (KeyGEN BioTECH, Nanjing, China) with 10% FBS (GIBCO) and 1% penicillin/streptomycin at 37°C and 5% CO_2_. All cell lines were negative for mycoplasma.

### Plasmid construction

The oligonucleotide 5′-GAAGAAAGUUCGUGGCAAA-3′ was used for construction of sh-OGT plasmids. And shRNA negative control was 5′- GTCAGGCTATCGCGTATCG-3′ (40). For knockdown of OGT, and the silencing plasmids containing shRNA sequences were constructed based on psilencer3.0-H1. The OGT overexpressing plasmid was obtained from WZ Biosciences, Inc. ORF of human OGT was constructed in pENTER vector, with C terminal Flag and His tag. All plasmids were verified by sequencing.

### Transfection

The miR-140 mimics and negative control (NC) were obtained from RIBOBIO company (Guangzhou, China). HighGene transfection reagent (Abclonal, Wuhan, China) was used to transfect the mimics into the PC3 and DU145 cells according to protocol. After 48 hours transfection, we then collected the cells for the next experiments.

### Apoptosis assay

Apoptosis rates were assessed using the Annexin V-FITC/PI Apoptosis Detection Kit (KeyGEN BioTECH, Nanjing, China) according to the instructions from the manufacturer. The cells were seeded into 6-well tissue culture plates (4 × 10 (5) cells/well). Following treatment, the cells were collected, washed with PBS, and resuspended in 500 μL binding buffer. Then, 5 μL Annexin V-FITC and 5 μL PI were added to the buffer and incubated at room temperature for 15 min in the dark. Cells were analyzed by flow cytometry (BD FACSCanto) within 1 hour.

### Drug sensitivity assay

Cells were seeded into 96-well plates with 3000 cells per well for at least three parallel experiments. 24 hours later, cells were exposed to increasing concentrations of docetaxel for 48 hours. Cell viability was assayed using the Cell Counting Kit-8 assay (SinoMol, Nanjing, China) according to the manufacturer’s instructions. At least three replicate experiments for each clone were averaged. Data are expressed as the percentage of growth relative to untreated controls.

### Luciferase reporter assay

The 3’ untranslated region (UTR) of OGT was cloned into the pMIR-Report luciferase vector. HighGene transfection reagent was used to transfect the vector into the cells. H293 cells at a density of 1 × 10 (5) per well in 24-well plates were co-transfected with wild-type or mutant vector with or without miR-140 mimics precursor, or scrambled oligonucleotide (RiBOBIO company, Guangzhou, China). Forty-eight hours after transfection, we used the dual luciferase reporter assay system to measure the activity of luciferase (Beyotime biotechnology, Shanghai, China).

### RNA extraction and quantitative PCR analysis

Total RNA was extracted using TRIzol reagent (Invitrogen) following the manufacturer’s protocol. For mRNA expression quantification, 1 μg of total RNA was converted to cDNA using the HiScript Q RT SuperMix for qPCR (Vazyme, Nanjing, China). RT-qPCR was performed using SYBR Green (cat. no. Q341-02; Vazyme, Nanjing, China) and operated on an ABI StepOne PCR system. Quantitative PCR primers sequences are listed in **Supplementary Table S1**.

### Western blot analysis

Western blot analysis was performed following the standard protocol. Total cellular protein was extracted using RIPA lysis buffer (P0013B; Beyotime Biotechnology), which contained 50 mM Tris (pH 7.4), 150 mM NaCl, 1% Triton X-100, 1% sodium deoxycholate and 0.1% SDS. And then protein was quantified using BCA protein assay reagent (Thermo, Shang, China). The primary antibodies used in this study are shown in **Supplementary Table S2**.

### Statistical analysis

Statistical analysis was conducted using GraphPad Prism 8.0 (GraphPad Software, Inc.). Data were analyzed using the Kolmogorov-Smirnov test for hypothesis of normal distribution and Bartlett’s test for homogeneity of variances. The statistical evaluation was performed by unpaired two-tailed Student’s t-test between two groups and one-way analysis of variance (ANOVA) among multiple groups. Tukey’s test was employed to assess the *post hoc* comparisons of different variables between multiple groups. Statistical significance was assumed when p < 0.05. All experiments were repeated at least three independent times. All data are displayed as the mean ± SD.

## Results

### OGT is overexpressed in PC cells and associated with poor survival

**Figure 1:**
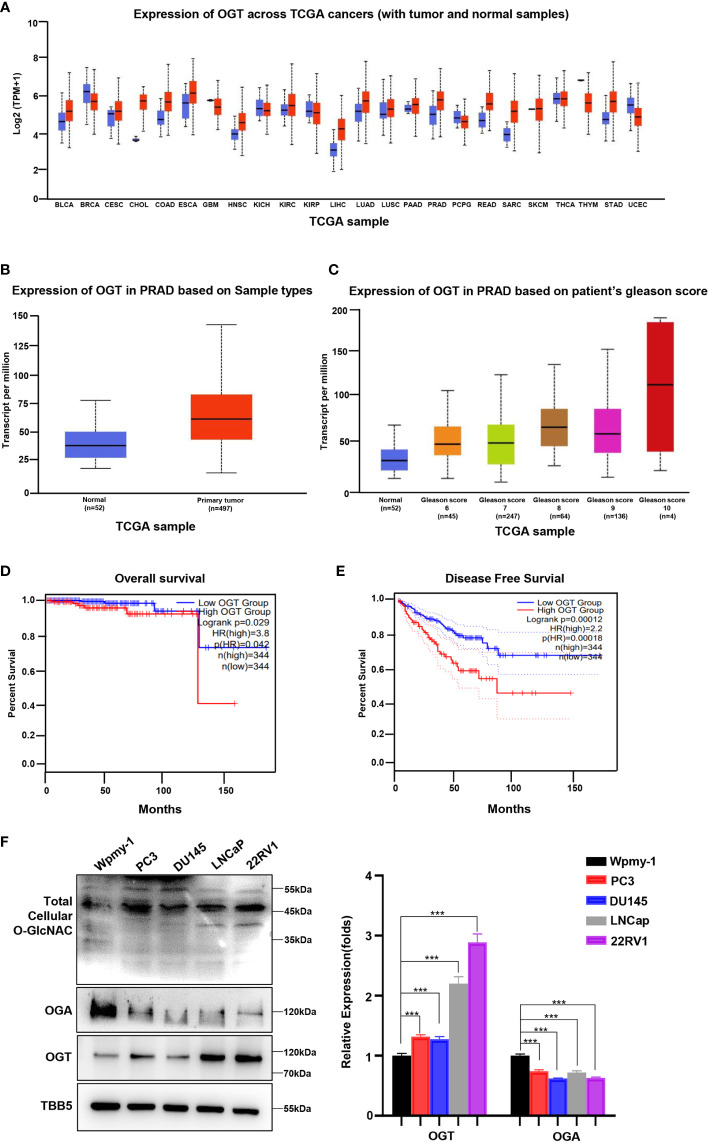
OGT is overexpressed in PC and contributes poor diagnosis in PC patients. **(A)** The expression level of OGT in different kinds of cancers and normal tissues from TCGA patient samples. **(B)** The expression level of OGT in primary prostate cancer tissues (n=497) and normal tissues(n=52) from TCGA patient samples. **(C)** OGT expression based on Gleason score from TCGA samples. **(D)** Kaplan–Meier analysis of the overall survival of patients with prostate cancer. The cut off value is 0.3. **(E)** The disease-free survival analysis of prostate cancer patients with high or low OGT expression from TCGA. The cut off value is 0.3. **(F)** Protein levels of OGT, OGA and total cellular O-GlcNAc in AR-positive (LNCaP, 22RV1) and AR-negative (PC3, DU145) PC cell lines and normal prostatic matrix immortalized cell (wpmy-1). *p < 0.05; **p < 0.01; ***p < 0.001 (Student’s t-test).

Abnormal expression of OGT has been showed to be related to the occurrence and development of several cancer types, including PC, breast cancer, colorectal cancer and other malignant tumors (26, 31, 41, 42). To explore the potential of OGT as a therapeutic target in PC, we firstly explored OGT expression patterns in publicly available data from the Cancer Genome Atlas (TCGA) on UALCAN website (http://ualcan.path.uab.edu/cgi-bin/Pan-cancer.pl?genenam=OGT). The results showed that OGT was dysregulated in many cancer types, with OGT being significantly higher in PC tissues\ (n=497) than in normal tissues (n=52) (**Figures 1A, B**). And the data from Gene Expression Omnibus (GEO) remains consistent (**Supplementary Figure S1**). Patients with higher Gleason score had higher OGT expression than that with lower Gleason score samples (**Figure 1C**). Gleason grade is the most important grading method for PC, which is mainly based on morphology/histology/pathology and compares the extent of cancer progression to normal tissue. The Gleason scoring system is the most frequently used grading system and uses a scale of 1 to 5 which determines the pattern of cell growth of the tumor. The specific assessment of cancer cell growth areas is assessed on a scale between 2 and 10 which is then adjusted to the scale of 1–5. A Gleason score of 7 refers to a medium-grade cancer, and a score of 8–10 refers to a high-grade cancer (43). Furthermore, Kaplan-Meier analyses were performed using data from GEPIA (http://gepia2.cancer-pku.cn/#survival), a tool for interactive analysis of gene expression profiles, providing differential expression analysis, profiling, correlation analysis and patient survival analysis based on TCGA and GTEx data (44). The results showed that PC patients with higher expression of OGT had a tendency of worse prognosis and shorter disease-free survival, compared with those with lower expression of OGT (**Figures 1D, E**). As shown in **Figure 1F**, higher OGT protein and O-GlcNAc levels were detected in both AR-positive (LNCaP, 22RV1) and AR-negative (PC3, DU145) PC cell lines, whereas OGA showed a lower expression, compared to those in human normal prostatic matrix immortalized cell wpmy-1.

### Knock-down of OGT sensitizes PC cells to docetaxel

**Figure 2:**
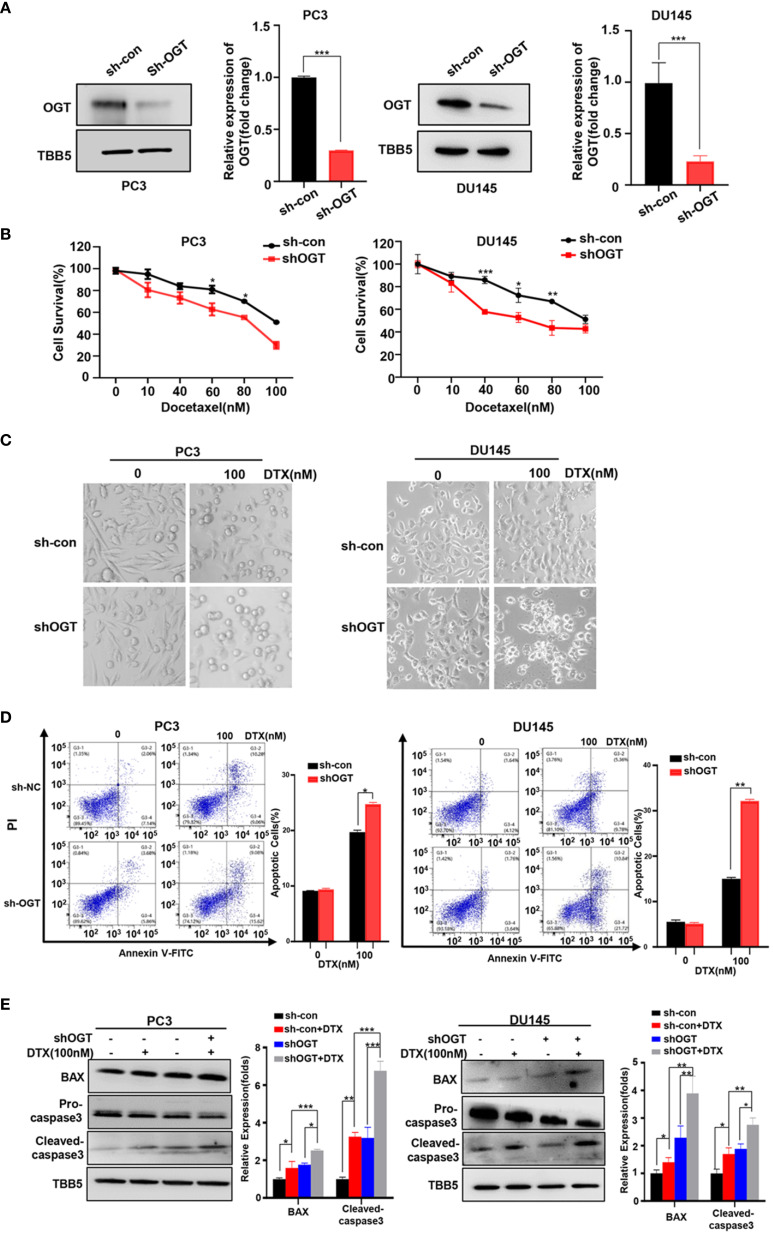
Knockdown of OGT sensitizes PC3 and DU145 cells to docetaxel. **(A)** The down-regulation of OGT and O-GlcNAC by sh-OGT plasmids was detected by Western blotting. **(B)** CCK8 assays using OGT-KD stable cell lines treated with docetaxel in PC3 and DU145. Data are expressed as the mean ± standard deviation (SD), n=3 per group. **(C)** Morphological analysis of control and OGT-KD cells treated with different doses of docetaxel. **(D)** Annexin V/PI staining and flow cytometry assay of control or OGT-KD cells with different drug treatments. **(E)** Western blot (WB) analysis of BAX and caspase-3 in control or OGT-KD PC3 and DU145 cells treated with various concentrations of docetaxel. All statistical data are presented as the mean ± SD. *p < 0.05; **p < 0.01; ***p < 0.001 (Student’s t-test).

In 2004, docetaxel was used as a first-line chemotherapy agent for CRPC to improve patient survival (45, 46). Docetaxel binds to β subunits of tubulin in microtubules, inhibits their depolymerization, blocks mitosis and ultimately induces apoptosis (47). However, docetaxel-induced side effects and acquired resistance limited its clinical efficacy. Thus, we wondered the effect of OGT inhibition on the sensitivity of PC cells to docetaxel. Firstly, OGT was knocked down by sh-OGT shRNA plasmid in PC cells (**Figure 2A**). As expected, O-GlcNacylation levels in whole cell lysates were significantly reduced in OGT-knocking down cells (**Figure 2A**). Then, CCK-8 assay was performed to detect cell survival under docetaxel treatment. PC3 and DU145 cells were transfected with sh-OGT or sh-Con and then treated with various concentrations of docetaxel (0, 10, 40, 60, 80, 100nM) for 48 hours. As shown in **Figure 2B**, knocking down OGT (sh-OGT) made cells more sensitive to docetaxel. The result was further verified in a morphological analysis (**Figure 2C**). Furthermore, flow cytometry analysis demonstrated that treatment with docetaxel induced higher apoptosis (Q2 late apoptosis and Q4 early apoptosis) rates in both sh-OGT PC3 and DU145 cells compared with control sh-Con cells (**Figure 2D**). The expression of pro-apoptotic protein BAX and cleaved caspase-3 shear band in OGT-knocking down PC3 and DU145 cells was higher than those in the control group after treatment with docetaxel (**Figure 2E**). The results suggested that the repression of OGT could elevate the sensitivity of PC cells to docetaxel. By contrast, overexpressing OGT could significantly reduce the drug sensitivity of PC cells to docetaxel, which was verified by cell survival assay and was (**Supplementary Figures S2A–C**).

### Small molecule inhibition of OGT sensitizes PC cells to docetaxel

**Figure 3:**
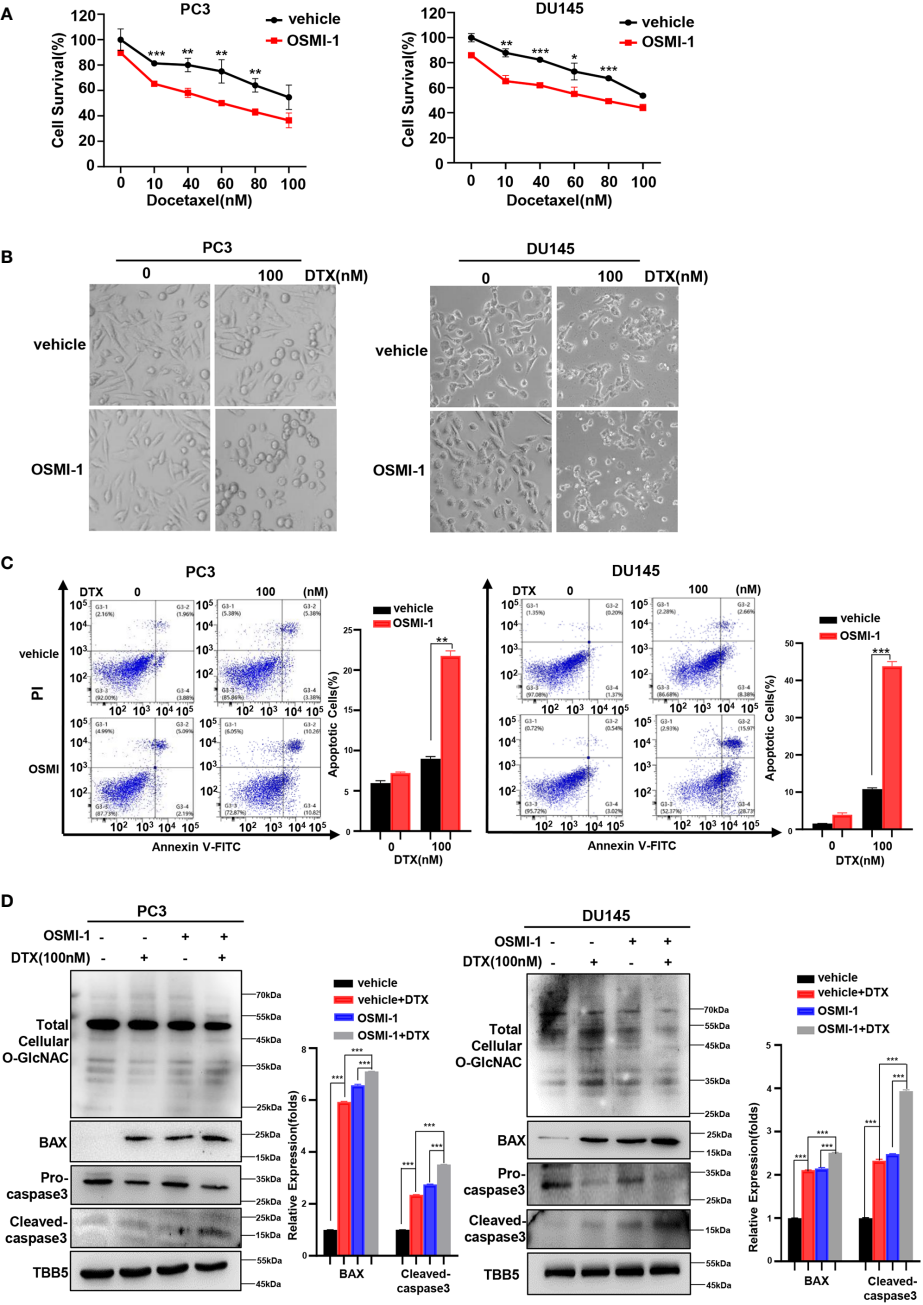
OSMI-1inhibition of OGT sensitizes PC cells to docetaxel. **(A)** Cell survival analysis in PC3 and DU145 cells with different concentration of OSMI-1 treatment. **(B)** Morphological analysis of PC3 and DU145 cells treated with OSMI-1, compared with control group. **(C)** Cell apoptosis was analyzed by flow cytometry. **(D)** Treatment with OSMI-1 enhanced the expression of pro-apoptotic proteins caspase-3 and BAX, and down-regulated the O-GlcNAc levels in PC3 and DU145 cells, compared with control group. *p < 0.05; **p < 0.01; ***p < 0.001 (Student’s t-test).

The combination of chemotherapy with chemo-sensitizing agents is a common approach to enhance anticancer activity while reducing the dose-dependent adverse side effects of cancer treatment (48). OGT inhibitors with some cellular activity have been reported, but most are substrate analogs that offer limited opportunities for modifications to improve potency or selectivity (32, 49, 50). OSMI-1 is a cell permeable molecule which potently inhibits OGT enzyme and in turn leads to an effective reduction in O-GlcNAcylation (32). Next, we investigated whether docetaxel combined with the OGT inhibitor, OSMI-1, was more cytotoxic to PC cells. Firstly, we detected the IC50 of OSMI-1 in PC3 and DU145 cell lines by CCK8 method, and then used a much lower dose (20 μM, one third of the IC50 value) for the combination with docetaxel in our further assay (**Supplementary Figure S3**). As shown in **Figure 3A**, cell viability was lower in the combination group than in the control group. The result was further verified in a morphological analysis, which showed more apoptotic cells indicating that combined treatment of docetaxel and OSMI-1 enhanced chemotherapeutic drug sensitivity (**Figure 3B**). We further confirmed whether OSMI-1 treatment enhanced the sensitization of PC3 and DU145 cells to docetaxel-induced apoptosis by flow cytometry. As shown in **Figure 3C**, flow cytometry confirmed that co-treatment with OSMI-1 induced a higher rate of apoptosis. Also, we showed higher expression of pro-apoptotic protein BAX and cleaved caspase-3 in the co-treatment group, compared with treatment with docetaxel alone (**Figure 3D**). As expected, the OSMI-1 treatment significantly reduced the expression of O-GlcNacylation levels in PC cells.

**Figure 4:**
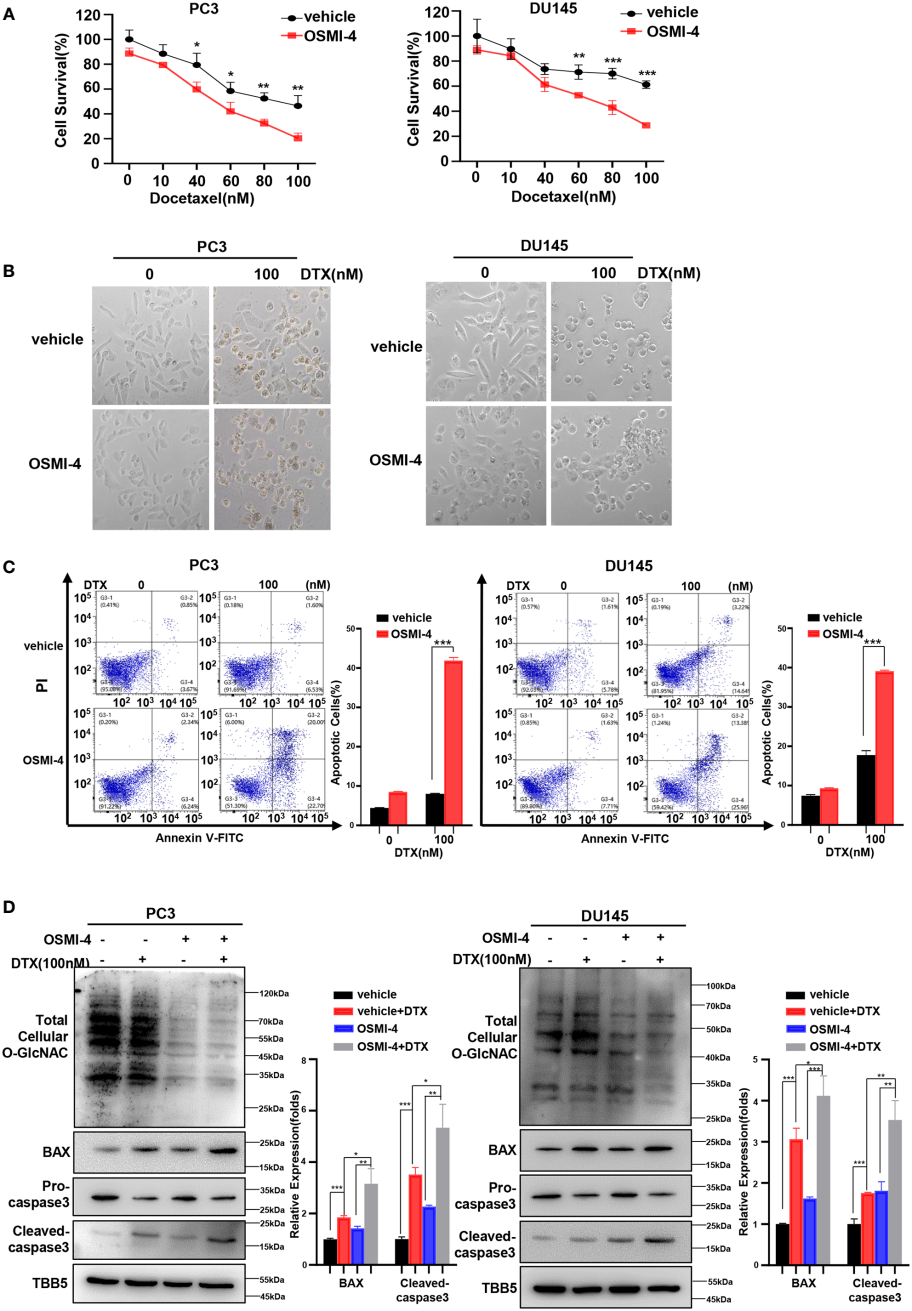
OSMI-4 inhibition of OGT sensitizes PC cells to docetaxel. **(A)** Cell survival analysis in PC3 and DU145 cells with different concentration of OSMI-4 treatment. **(B)** Morphological analysis of PC3 and DU145 cells treated with OSMI-4, compared with control group. **(C)** Cell apoptosis was analyzed by flow cytometry. **(D)** Treatment with OSMI-4 enhanced the expression of pro-apoptotic proteins caspase-3 and BAX, and down-regulated the O-GlcNAc levels in PC3 and DU145 cells, compared with control group. *p < 0.05; **p < 0.01; ***p < 0.001 (Student’s t-test).

Recently, a series of potent OGT inhibitors based on a previous hit (OSMI-1) were synthesized and evaluated in the Walker laboratory (Harvard University, Boston, MA, USA). OSMI-4 is the best OGT inhibitor reported to date, with a ~3 μM EC50 in cells, making it especially attractive for probing OGT’s complex biology (51). Thus, we further detected the effect of combination of OSMI-4 and docetaxel on anti-tumor ability. We first detected the IC50 of OSMI-4 in PC3 and DU145 cell lines by CCK8 method (**Supplementary Figure S4A**). The changes of O-GlcNacylation levels in PC3 and DU145 cell lines under different concentrations of OSMI-4 treatment were detected by western blot. As shown in **Supplementary Figure S4B**, OSMI-4 reduced O-GlcNAc levels almost completely by 9 μM in PC3 and DU145 cells. Then we used 9 μM for the combination with docetaxel in our further assay. CCK8 assay results showed that both PC3 and DU145 cells under OSMI-4 treatment were more sensitive to docetaxel (**Figure 4A**). The results were further verified in a morphological analysis, which showed more apoptotic cells indicating that combined treatment of docetaxel and OSMI-4 enhanced chemotherapeutic drug sensitivity (**Figure 4B**). Subsequently, we examined the effect of the combination of OSMI-4 and docetaxel on apoptosis by flow cytometry. As shown in **Figure 4C**, flow cytometry confirmed that co-treatment with OSMI-4 induced a higher rate of apoptosis. While the OSMI-4 treatment significantly reduced the O-GlcNacylation levels, higher expression of pro-apoptotic protein BAX and cleaved caspase-3 were obtained in the co-treatment PC cells, compared with those under the treatment with docetaxel alone (**Figure 4D**). The above experimental results demonstrated that pharmacologic inhibition of OGT increased the sensitivity of PC cells to docetaxel and induced stronger apoptosis.

### MiR-140 targets and represses OGT expression

The regulation of OGT has been intensively studied and recently, it has been shown that in addition to those traditional factors, miRNAs also play an important role. To figure out potential miRNAs regulating OGT expression, we performed bioinformatics analysis using Target Scan (https://www.targetScan.org/vert_80/) (52, 53), which identified nine miRNAs potentially binding and targeting to the 3′ UTR region of OGT (**Supplementary Table S3**). Subsequently, Pic Tar (https://pictar.mdc-berlin.de/) (54), miRcode (http://www.mircode.org/) (55) and Starbase databases (https://ngdc.cncb.ac.cn/databasecommons/database/id/169) (56, 57) were used to analyze the binding potential of these miRNAs to OGT, with miR-140 and miR-24-3p having the highest overall scores (**Supplementary Table S3**). Numerous studies have reported that miR-140 serves as a tumor suppressor in several types of cancers (58, 59). Our previous study found that miR-140 inhibits Flap endonuclease 1 (FEN1) expression *via* directly binding to its 3′ untranslated region, leading to impaired DNA repair and inhibition of breast cancer progression (60). MiR-140 aggravates doxorubicin-induced cardiotoxicity by promoting myocardial oxidative stress *via* targeting Nrf2 and Sirt2 (61). Zhao et al. reported that miR-140 inhibited PC cells invasion and migration by targeting YES proto-oncogene 1, indicating that miR-140 could be a potential target for PC therapy (62).

**Figure 5:**
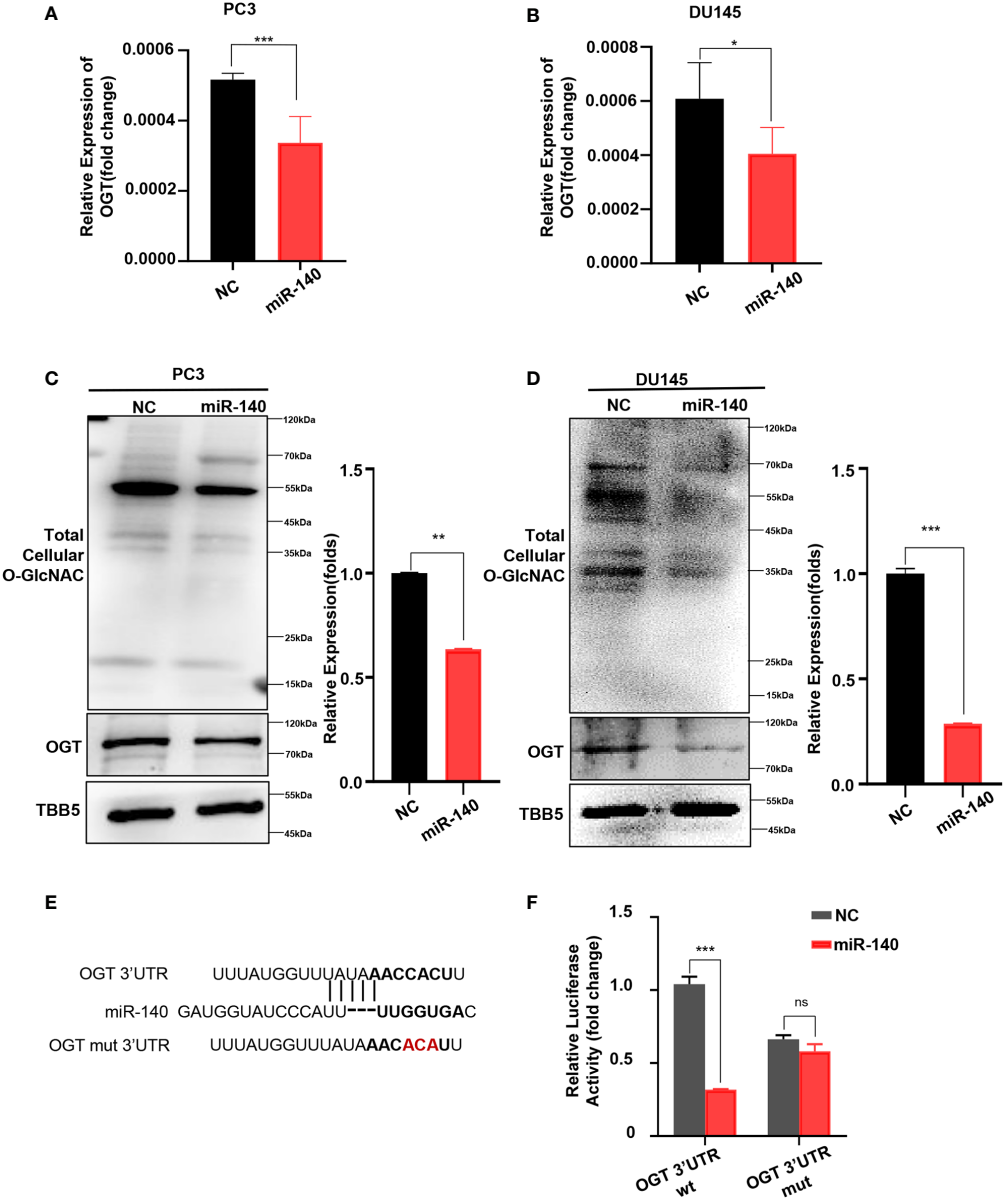
miR-140 targets and represses OGT expression. **(A, B)** Overexpression of miR-140 represses the *OGT* mRNA levels in PC3 and DU145 cells. NC: Negative control. **(C, D)** Overexpression of miR-140 represses the OGT protein expression and O-GlcNAC levels in PC3 and DU145 cells. **(E)** The putative miR-140 binding sequence in the 3′UTR of OGT. Red, mutations of the miR-140 binding site in the OGT 3′UTR. **(F)** Analyses of the luciferase activity of the luciferase reporter plasmids containing either wild-type (WT) or mutant-type (MT) OGT 3′UTRs in HEK-293 cells. *p < 0.05; **p < 0.01; ***p < 0.001 (Student’s t-test).

Next, we explored whether miR-140 directly affects OGT expression in PC. First, we transfected miR-140 mimics into PC3 and DU145 cell lines and verified the transfection efficiency by RT-qPCR (**Supplementary Figure S5**). And then we examined the effect of miR-140 on the mRNA and protein levels of OGT. Our data showed that overexpression of miR-140 using miRNA mimics significantly downregulated the *OGT* mRNA in PC3 cells (**Figure 5A**) and DU145 cells (**Figure 5B**). Overexpression of miR-140 also downregulated OGT protein expression and O-GlcNAc levels (**Figures 5C, D**) in both cell lines. We predicted the binding sites of miR-140 to OGT gene in the Target Scan database and identified potential binding sites for the 3 ‘UTR. To further identify, we performed luciferase reporter assays and mutagenesis analyses. We constructed luciferase reporter plasmids: pMIR-OGT-3’UTR-wt, containing pairing sites for miR-140 in the OGT-3’UTR, and pMIR-OGT-3’UTR-mut, containing the mutated OGT-3’UTR (**Figure 5E**). We found that the luciferase activity of the wild-type (WT) plasmid was significantly attenuated by miR-140, whereas the activity of the mutant plasmid was not. (**Figure 5F**). These results indicated that miR-140 regulated OGT by directly targeting its 3′UTR.

### Docetaxel treatment decreases OGT expression and increases miR-140 expression

**Figure 6:**
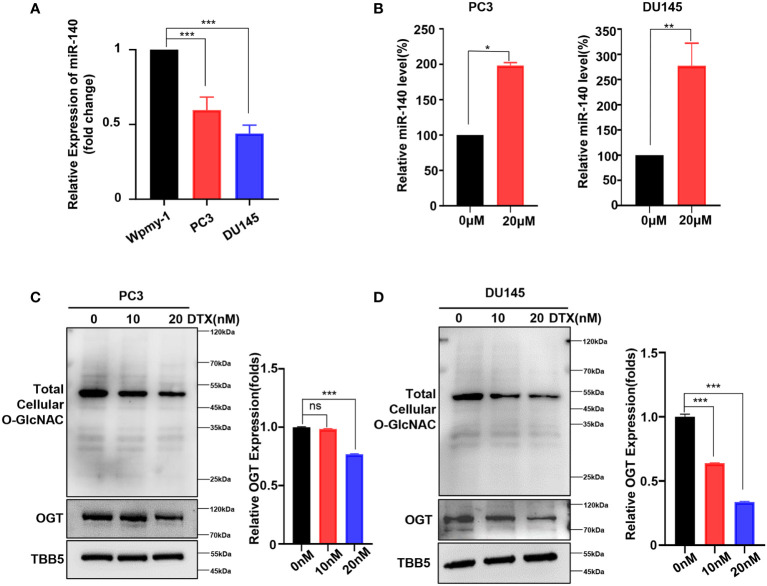
Docetaxel treatment decreases OGT expression and increases miR-140 expression. **(A)** RT-qPCR results showed that miR-140 expression levels were significantly downregulated in PC cell. Values are expressed as the mean ± standard deviation. **(B)** MiR-140 expression levels were upregulated with the treatment of docetaxel. **(C, D)** OGT protein and O-GlcNAc levels were dramatically downregulated in PC3 and DU145 cells treated with different concentrations of docetaxel. *p < 0.05; **p < 0.01; ***p < 0.001 (Student’s t-test). ns, means no significance.

As mentioned above, we showed that genetic knockdown of OGT or pharmacologic inhibition of OGT all increased the sensitivity of PC cells to docetaxel. Next, we wondered whether this chemotherapeutic drug had an effect on miR-140 and OGT expression. The expression of miR-140 in PC cell lines was measured by RT-qPCR analysis. As showed in **Figure 6A**, miR-140 was down-regulated in PC3 and DU145 cells, compared to the normal prostate epithelial cell line. Interestingly, miR-140 expression was significantly increased in PC3 and DU145 cells treated with docetaxel (**Figure 6B**). In contrast, while OGT is significantly upregulated in PC cells (**Figure 1F**), docetaxel treatment downregulated OGT and O-GlcNAc expression in both PC3 and DU145 cells (**Figures 6C, D**). However, the *OGT* mRNA levels were not significantly down-regulated after docetaxel treatment (**Supplementary Figure S6**).

### Overexpression of miR-140 enhances the sensitization of PC cells to docetaxel

**Figure 7:**
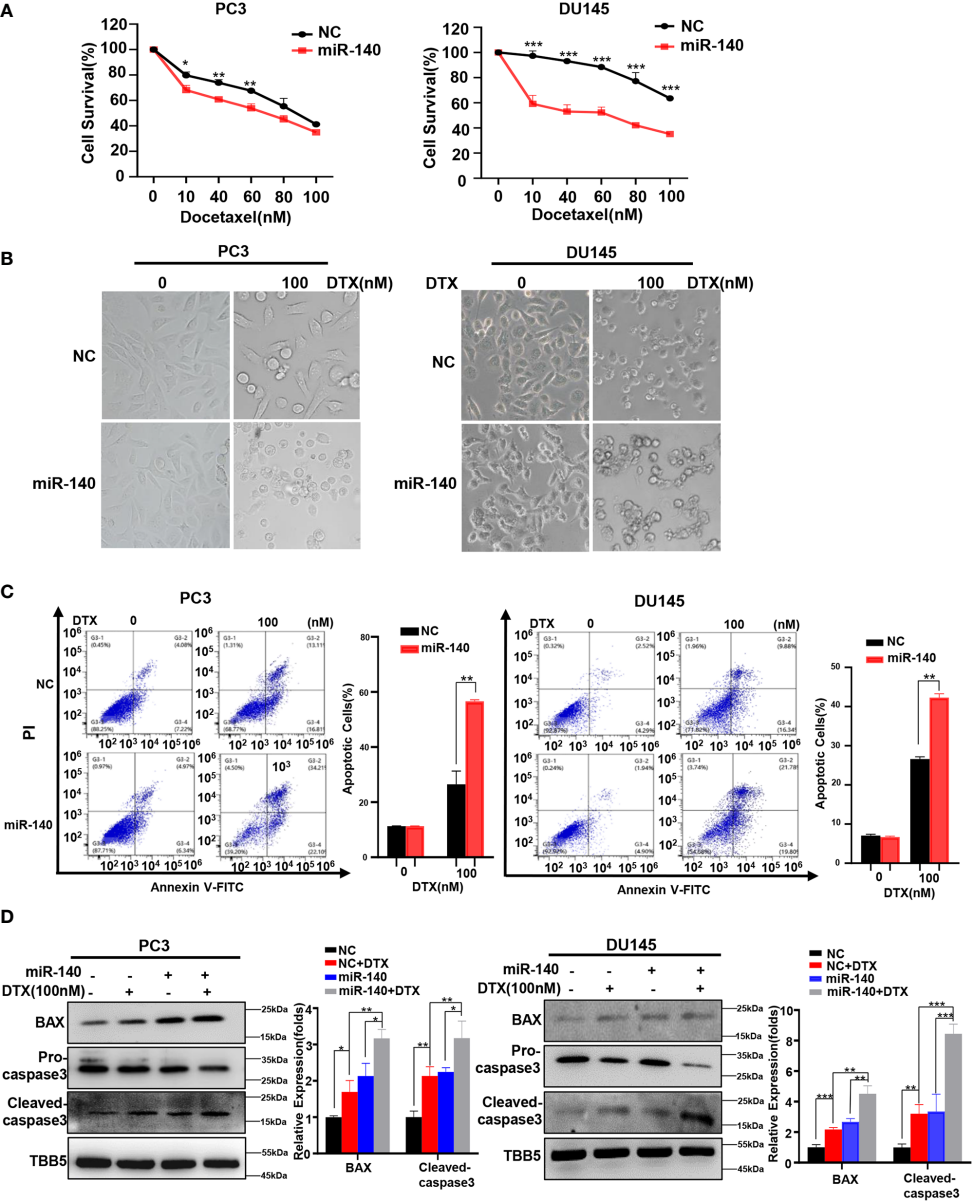
Overexpression of miR-140 enhances the sensitization of PC cells to docetaxel. **(A)** CCK8 assays using miR-140-overexpressing stable cell lines treated with docetaxel in PC3 and DU145. Data are expressed as the mean ± standard deviation (SD), n=3 per group. **(B)** Morphological analysis of control and miR-140-overexpressing cells treated with different doses of docetaxel. **(C)** Cell apoptosis was analyzed by flow cytometry. **(D)** Western blot (WB) analysis of BAX and caspase-3 levels in control or miR-140-overexpressing PC3 and DU145 cells treated with various concentrations of docetaxel. All statistical data are presented as the mean ± SD. *p < 0.05; **p < 0.01; ***p < 0.001 (Student’s t-test).

We previously found that miR-140 enhanced chemotherapeutic response in breast cancer cells (60). We wondered whether miR-140 overexpression also increased the sensitivity of PC cells to docetaxel. Cell viability assays using miR-140-overexpressing PC3 and DU145 stable cell lines treated with serial concentrations of docetaxel (10, 40, 60, 80 and 100 nM) revealed that overexpression of miR-140 sensitized PC3 and DU145 cells to docetaxel (**Figure 7A**). The result was further verified in morphological analysis (**Figure 7B**). In addition, flow cytometry analysis demonstrated that treatment with docetaxel induced higher apoptosis rates in both miR-140 overexpression PC3 and DU145 cells compared with control cells (**Figure 7C**). The expression of BAX and cleaved caspase-3 was higher in miR-140-overexpressing PC3 and DU145 cells than in the control group after treatment with doxorubicin (**Figure 7D**).

### MiR-140-induced PC cells sensitization to DTX is OGT dependent

**Figure 8:**
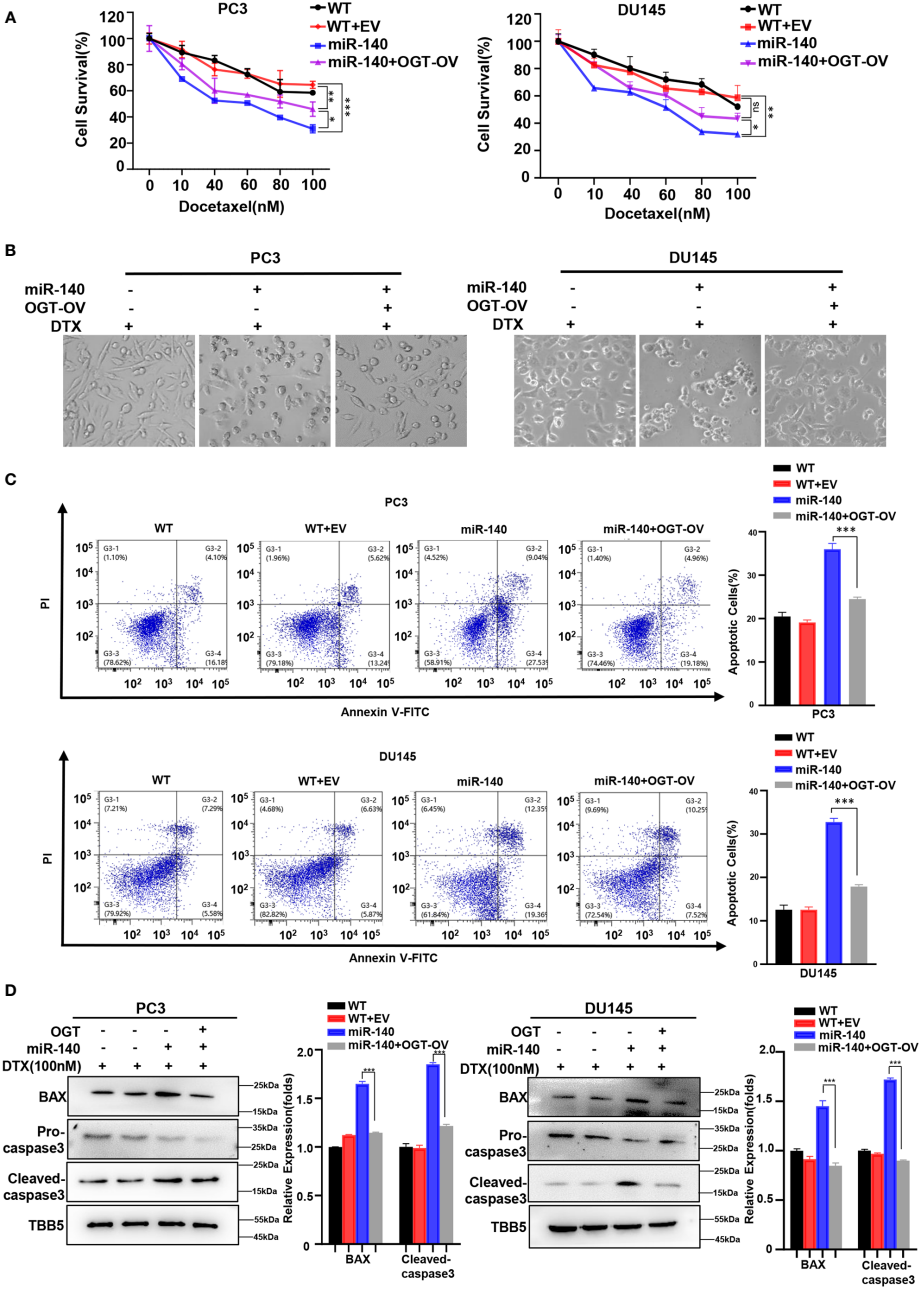
MiR-140-induce PC cells sensitization to DOCETAXEL is OGT dependent. **(A)** CCK8 revealed that overexpression of miR-140 enhanced the sensitivity of cells to docetaxel, which was rescued by overexpression of OGT. **(B)** Morphological analysis of different treated PC3 and DU145 cells. **(C)** MiR-140 increased docetaxel-induced cell apoptosis, which was rescued by overexpression of OGT. **(D)** Western blot(WB) analysis of BAX and caspase-3 levels in PC3 and DU145 cells overexpressing OGT with miR-140.

We next examined whether miR-140 sensitized PC cells to docetaxel by targeting and inhibiting OGT. Our data showed that under the same concentration gradient of docetaxel treatment, the viability of miR-140-overexpressing cells was significantly reduced compared to the control cells, which was consistent with previous results (60, 63). By contrast, OGT overexpression reduced the drug sensitivity of PC cells (**Figure 8A**). Similarly, morphological experimental results showed that overexpression of OGT could abrogate the sensitizing effect of miR-140 to docetaxel (**Figure 8B**). Compared with miR-140-overexpressing cells, the apoptosis ratios decreased in the miR-140/OGT double overexpressing cells (**Figure 8C**). Moreover, the expression of pro-apoptotic proteins BAX and cleaved caspase3 was down-regulated in the miR-140/OGT-overexpressing group compared to miR-140-overexpressing cells (**Figure 8D**). Accordingly, overexpression of OGT reduced the levels of apoptosis in miR-140-overexpressing PC cells. These results indicated that miR-140 augmented chemotherapeutic sensitivity in PC treatment by at least partially targeting OGT.

## Discussion

O-GlcNAcylation is critical in regulating cancer development and progression. In recent years, the function of O-GlcNAcylation in tumors has received extensive attention because of its key role in promoting tumor growth and migration. Previous studies showed that high expression of OGT was related to drug resistance and poor prognosis of several kinds of cancers (12, 64). Therefore, inhibition of OGT is a strategy that can provide a benefit to chemotherapy by sensitizing cancer cells to anticancer agents or overcoming drug resistance in cancer (65). The study by Harri M. Itkonen et al. highlights the potential of OGT inhibitors in combination with other therapies to benefit cancer-specific vulnerabilities and reports a novel synthetic lethal interaction between OGT and CDK9 inhibitors (19). In addition, inhibitors targeting OGT and GPT2 identified synergistic lethal combinations specific for PC cells (31). In this study, we showed that OGT is high expressed in PC and associated with the poor prognosis in PC patients. Inhibition of OGT by chemical inhibitor could sensitize PC cells to DTX. Moreover, endogenous miR-140 could repress OGT by directly targeting 3’ UTR of OGT transcript, further enhancing the sensitization of PC cells to DTX. The effect of miR-140 on PC cells sensitization to DTX could be reversed by overexpression of OGT. Our study indicated a novel combinational strategy for PC chemotherapy.

Chemotherapy is the main form cancer treatment that aims to prolong life or reduce symptoms. Cytotoxic chemotherapy has been used to treat PC for several decades, but only taxanes have been shown to prolong OS in patients with advanced PC. In 2004, the Phase III clinical trials TAX327 and SWOG 99-16 demonstrated longer OS for patients with mCRPC treated with docetaxel versus mitoxantrone, thus making docetaxel the standard of care for chemotherapy-naïve mCRPC patients (8, 46). Our study found that inhibition of OGT by shRNA or OGT inhibitors, OSMI-1 and OSMI-4, made PC cells more sensitive to docetaxel. Under the same dose of drug treatment, cells with low O-GlcNAc levels showed lower survival rate, whereas higher apoptosis rate. However, docetaxel therapy is typically limited by development of tumor resistance, and efforts have been made to further understand the molecular mechanisms of resistance, and to develop new therapies to overcome them (66, 67). We found that OGT can be used as a target to enhance the sensitivity of prostate cancer cells to chemotherapy drugs, which might also play a role in drug resistance of PC cells. Although several compounds targeting cancer-specific metabolic abnormalities are currently assessed in clinical trials (68, 69), the OGT inhibitors are still not yet, including in PC. More rigorous toxicological and pharmacological testing may still need in animal and preclinical test. We also hope these inhibitors could be used in clinic treatment in the future soon.

Lifeng Ding et al. discuss the characteristics and vital role of noncoding RNAs, especially miRNA, lncRNA, and circRNA, in drug resistance of PC. These noncoding RNAs are potential therapeutic targets for treating drug resistance in prostate cancer (70, 71). Since noncoding RNAs play significant roles in tumorigenesis, more and more attention has been focused on the relationship between noncoding RNA and chemoresistance (70). Our previous study demonstrated that miR-140 enhanced the chemotherapeutic response in breast cancer cells to doxorubicin (60). However, the relationship between miR-140 and chemotherapy in PC remains unclear. In this study, we demonstrated that overexpression of miR-140 sensitizes PC cells to docetaxel, while overexpression of OGT inhibited the effect of miR-140.

In most cases, miRNAs are regarded to be negative regulators of gene expression (72). Previous studies indicated that miR-140 functions as a tumor suppressor by targeting several kinds of genes, including FEN1 (60), YES1 (62) and BIRC6 (73). Downregulation of miR-140 is implicated in the development of PC by regulating cancer stem cell formation, tumor invasion, and angiogenesis (73, 74). We used Target Scan database, Pic Tar, miRcode and Starbase databases to identify miR-140 as a potential miRNA targeting OGT. Indeed, we demonstrated that miR-140 repressed OGT expression by directly targeting its3′UTR. Furthermore, we found that miR-140 expression was significantly downregulated, whereas OGT expression was significantly upregulated in PC3 and DU145 cells compared with normal prostate tissue cells. Of note, miR-140 expression was significantly upregulated while OGT protein levels were downregulated in PC cells under docetaxel treatment. However, the *OGT* mRNA levels were not significantly down-regulated after docetaxel treatment. We speculate that the reduction in OGT protein expression in the presence of docetaxel could be partially due to caspase-mediated proteolysis during apoptosis. The slight increase of *OGT* mRNA level after docetaxel treatment may be due to a negative feedback regulation after the down-regulation of OGT protein level. These data suggest that miR-140 may influence the sensitivity of PC cells to chemotherapy drug docetaxel by regulating the miR-140/OGT axis, providing a new strategy for overcoming the resistance of tumor cells to chemotherapy drugs.

In conclusion, our findings demonstrated that inhibition of OGT enhanced the chemotherapeutic response in PC cells. Moreover, miR-140 directly targeted OGT and inhibited OGT expression, increasing docetaxel-induced apoptosis and enhancing the drug sensitivity of PC cells to docetaxel. This study provides a new strategy and potential targets for the treatment of PC.

## Data availability statement

The original contributions presented in the study are included in the article/**Supplementary Material**. Further inquiries can be directed to the corresponding author.

## Author contributions

MX, SW, YQ, KL, and EL performed study concept and design. MX, SW, ZH, KL, EL, FP and LH performed development of methodology and writing, review and revision of the paper. MX, SW, ZH and ZG provided acquisition, analysis and interpretation of data, and statistical analysis. ZH and ZG provided technical and material support. All authors read and approved the final paper.

## Funding

This work was supported by National Natural Science Foundation of China (32171407, 81872284) and the Priority Academic Program Development of Jiangsu Higher Education Institutions.

## Conflict of interest

The authors declare that the research was conducted in the absence of any commercial or financial relationships that could be construed as a potential conflict of interest.

## Publisher’s note

All claims expressed in this article are solely those of the authors and do not necessarily represent those of their affiliated organizations, or those of the publisher, the editors and the reviewers. Any product that may be evaluated in this article, or claim that may be made by its manufacturer, is not guaranteed or endorsed by the publisher.
