# Corrigendum: Inhibition of O-GlcNAc transferase sensitizes prostate cancer cells to docetaxel

**DOI:** 10.3389/fonc.2023.1257404

**Published:** 2023-07-31

**Authors:** Mingyue Xia, Shuyan Wang, Yannan Qi, Kaili Long, Enjie Li, Lingfeng He, Feiyan Pan, Zhigang Guo, Zhigang Hu

**Affiliations:** Jiangsu Key Laboratory for Molecular and Medical Biotechnology, College of Life Sciences, Nanjing Normal University, Nanjing, China

**Keywords:** OGT, chemotherapy, docetaxel, miR-140, prostate cancer

**Error in Figure/Table**

In the published article, there was an error in **Figure 2C** as published. The panel in the bottom left of the figure was misused due to an error in the final assembly of **Figure 2**. The corrected **Figure 2C** and its caption “Knockdown of OGT sensitizes PC3 and DU145 cells to docetaxel” appear below.

**Figure 2:**
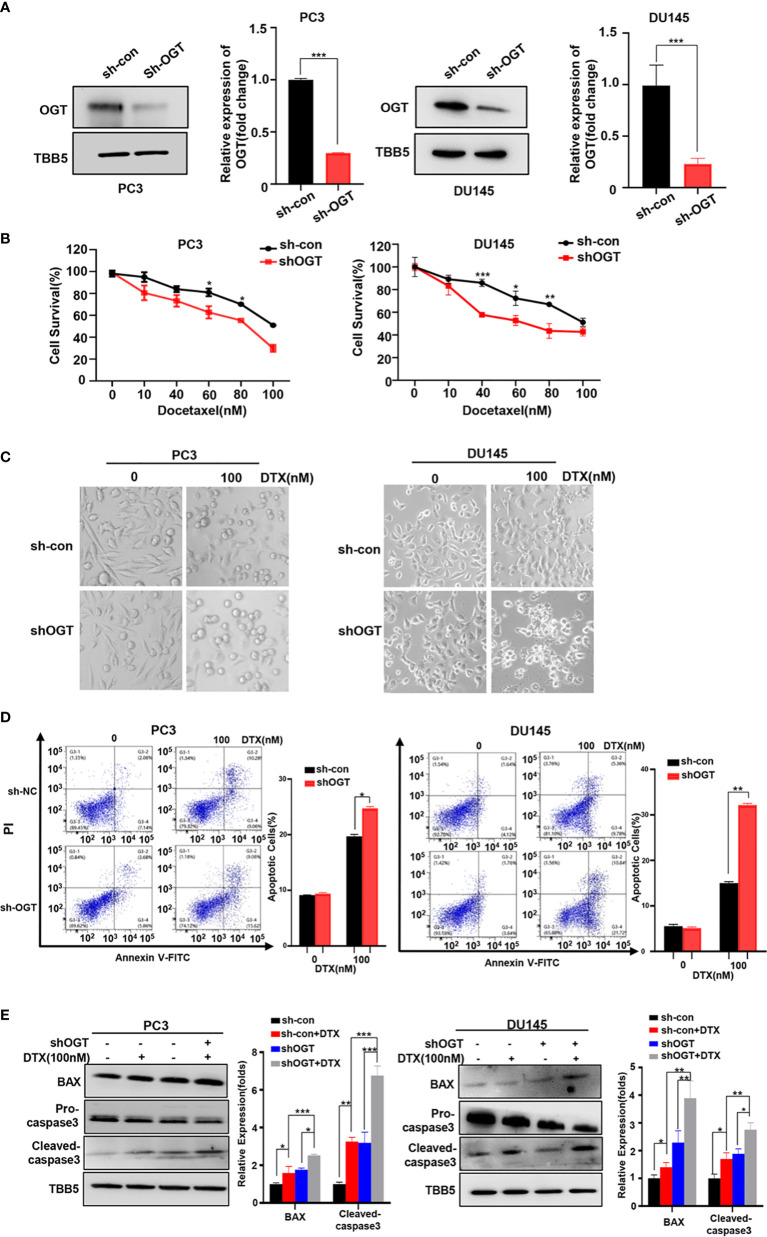
Knockdown of OGT sensitizes PC3 and DU145 cells to docetaxel. **(A)** The down-regulation of OGT and O-GlcNAC by sh-OGT plasmids was detected by Western blotting. **(B)** CCK8 assays using OGT-KD stable cell lines treated with docetaxel in PC3 and DU145. Data are expressed as the mean ± standard deviation (SD), n=3 per group. **(C)** Knockdown of OGT sensitizes PC3 and DU145 cells to docetaxel. **(D)** Annexin V/PI staining and flow cytometry assay of control or OGT-KD cells with different drug treatments. **(E)** Western blot (WB) analysis of BAX and caspase-3 in control or OGT-KD PC3 and DU145 cells treated with various concentrations of docetaxel. All statistical data are presented as the mean ± SD. *p < 0.05; **p < 0.01; ***p < 0.001 (Student’s t-test).

The authors apologize for this error and state that this does not change the scientific conclusions of the article in any way. The original article has been updated.

