# Supplementary material for: Inhibition of O-GlcNAc transferase sensitizes prostate cancer cells to docetaxel
Source: Front Oncol. 2022 Nov 10;12:993243. doi: 10.3389/fonc.2022.993243 (PMC9686342; doi:10.3389/fonc.2022.993243)
Supplement: Supplementary file 1 [file DataSheet_1.pdf]

Inhibition of O-GlcNAc transferase sensitizes prostate cancer cells to docetaxel

Mingyue Xia§, Shuyan Wang§, Yannan Qi§, Kaili Long, Enjie Li, Lingfeng He, Feiyan Pan, Zhigang Guo, Zhigang Hu*

[1]Jiangsu Key Laboratory for Molecular and Medical Biotechnology, College of Life Sciences, Nanjing Normal University, Nanjing210023, China

Supplementary Information

The following file contains supplementary material for the paper "Inhibition of O-GlcNAc transferase sensitizes prostate cancer cells to docetaxel".

This file is composed of:

Supplementary tables (3 tables)

Supplementary figures and relative supplementary figure legends (6 figures)

Supplementary table 1. Sequences of the primers used in this study.

Name	Oligonucleotide sequence	Application
OGT	F:CGCGGATCCGAAGCTCCAGATGGCGTCTTC	Primers used for QRT-PCR
	R:ATTTGCGGCCGCCATGCTGACTCAGTGACTTCAAC	
GAPDH	F:GTCTCCTCTGACTTCAACAGCG	
	R:ACCACCCTGTTGCTGTAGCCAA	
OGT	F:GATCCGGTTCTGCCTTCTGGACAATGTGCTTTTGTCCAGAAGGCAGAACCTTTTTA	Primers used for Plasmids construction
	R:CGCGTAAAAAGGTTCTGCCTTCTGGACAAAAGCACATTGTCCAGAAGGCAGAACCG	

Supplementary table 2. Antibodies used in this study.

Antibody	Source	Identifier	Additional information
Rabbit polyclonal anti-TBB5	ABclonal	Cat# A11577	WB(1:1000)
Monoclonal anti-BAX	Cell Signaling Technology	Cat# 80,312 S	WB(1:1000)
Ployclonal anti-caspase3	ABclonal	Cat# A11550	WB(1:1000)
Rabbit polyclonal anti-OGT	proteintech	Cat# 11576-2-AP	WB(1:1000)

Supplementary table 3. Analysis of miRNAs target to OGT 3' UTR.

1 and 0 represent the positive and negative results analysed by bioinformatics tools, respectively.

miRNAs	TargetScan	PicTar	miRcode	Starbase	SUM
miR-140-5p	1	1	1	1	4
miR-24-3p	1	1	1	1	4
miR-204-5p	1	1	0	1	3
miR-141-3p	1	1	0	1	3
miR-181-5p	1	1	0	1	3
miR-182-5p	1	1	0	1	3
miR-145-5p	1	0	0	1	2
miR-200a-3p	1	1	0	0	2
miR-202-5P	1	0	0	0	1

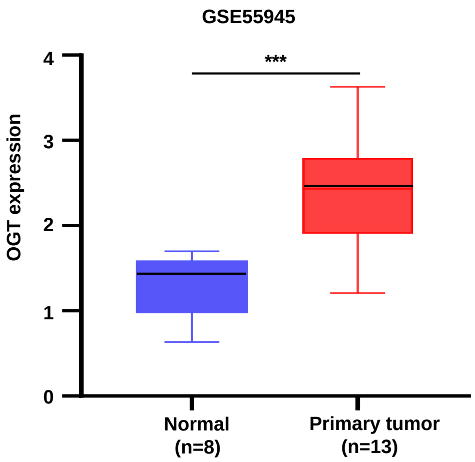

Supplementary Fig. 1. The relative expression level of OGT in publicly-available data from Gene Expression Omnibus (GEO). *** $p<0.001$ (Student's t-test).

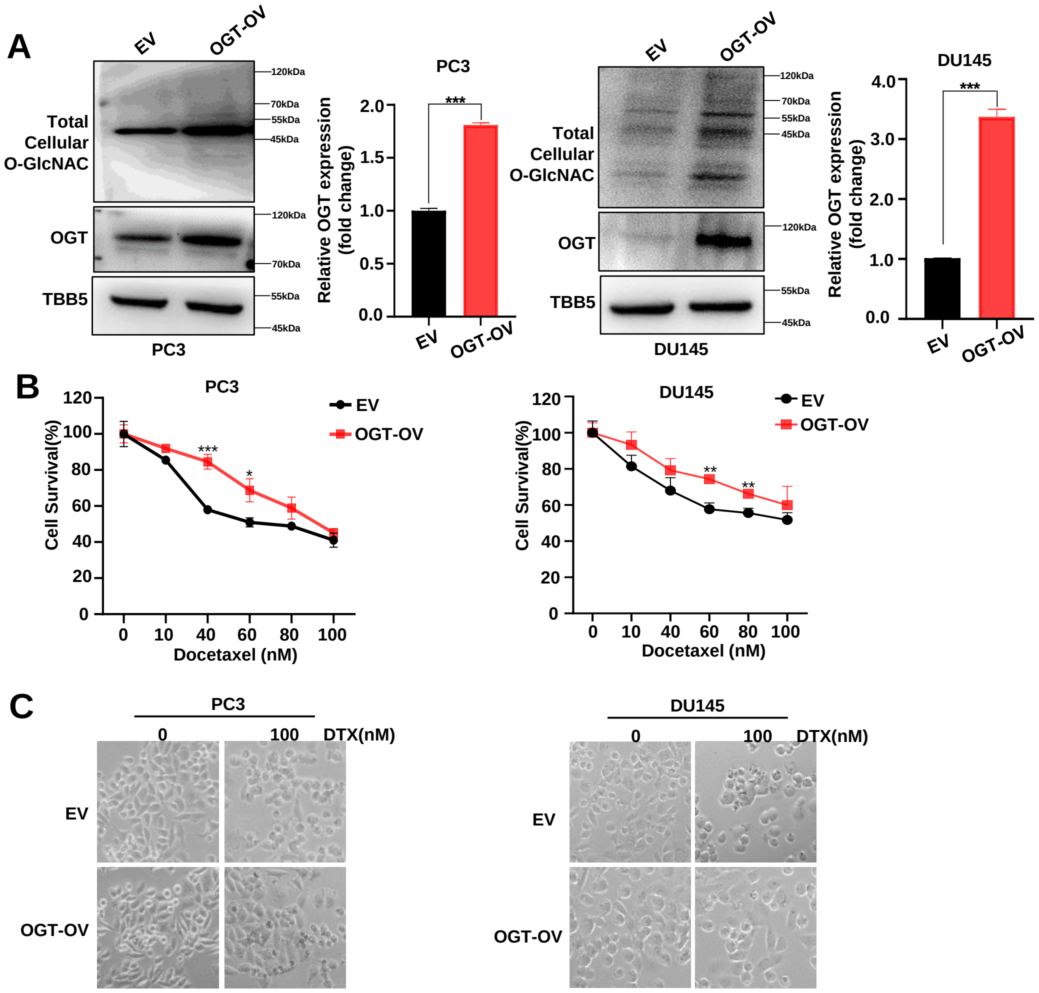

Supplementary Fig. 2. Overexpressing OGT could significantly reduce the drug sensitivity of PC cells to docetaxel.

(A) Western blot was used to validate of the up-regulation of OGT protein and O-GlcNAc levels. *** $p<0.001$ (Student's t-test). (B) CCK8 assays were used to detect docetaxel sensitivity in OGT-overexpressing PC3 and DU145 cell lines. Data are expressed as the mean ± standard deviation (SD), n=3 per group. (C) Morphological analysis of control and OGT-overexpressing cells treated with docetaxel.

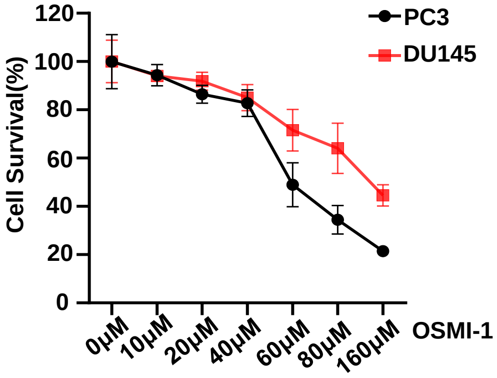

Supplementary Fig. 3. CCK8 assay to detect the IC50 of OSMI-1 in PC3 and DU145 cells.

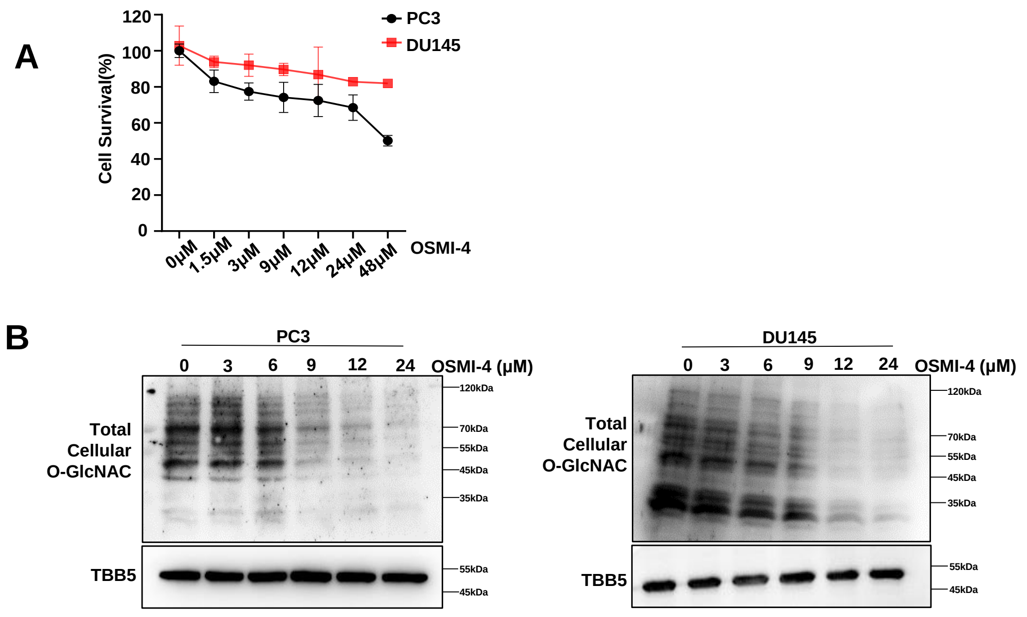

Supplementary Fig. 4. (A) CCK8 assay to detect the IC50 of OSMI-4 in PC3 and DU145 cells. (B) Western blot assays were used to detect the changes of O-GlcNacylation expression in PC3 and DU145 cell lines under different concentrations of OSMI-4 treatment.

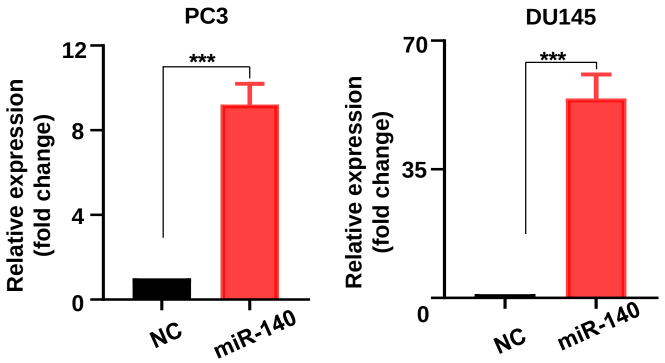

Supplementary Fig. 5. qRT-PCR was used to validate of the efficiency of miR-140 overexpression. *** p<0.001 (Student's t-test).

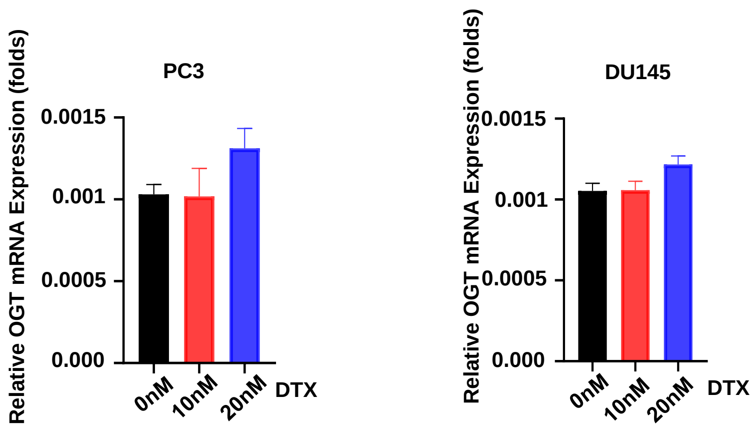

Supplementary Fig. 6. The mRNA expression levels of OGT in PC3 and DU145 cell lines under docetaxel treatment were detected by RT-qPCR.
